# The relationship between futile medical care and respect for patient dignity: a cross-sectional study

**DOI:** 10.1186/s12912-022-01144-1

**Published:** 2022-12-28

**Authors:** Faezeh Nazari, Maryam Chegeni, Parvin Mongolian Shahrbabaki

**Affiliations:** 1 MSc in Nursing, Afzalipour Hospital, Kerman University of Medical Science, Kerman, Iran; 2 Department of Public Health, Khomein University of Medical Sciences, Khomein, Iran, Molecular and Medicine Research Center, Khomein University of Medical Sciences, Khomein, Iran; 3 Nursing Research Center, Kerman University of Medical Sciences, Kerman, Iran

**Keywords:** Futile care, Intensive care unit, Nurses, Patient, Respect for patient dignity

## Abstract

**Background:**

Various technologies and interventions at intensive care units can lead to futile medical care for critically ill patients. Futile medical care increases patients’ suffering and costs, reduces nurses' attention to patients, and thus affects patients’ dignity. This study aimed to investigate the relationship between futile medical care and respect for patient dignity from the perspective of nurses working in intensive care units of medical centers.

**Methods:**

We conducted this cross-sectional study on 160 nurses working in intensive care units in Kerman. We measured nurses’ perceptions of futile care and respect for patient dignity using futile care and patients’ dignity questionnaire. We used linear regression model to investigate the effect of futile care on the patient dignity.

**Results:**

The mean severity and frequency of futile care in the intensive care unit were 57.2 ± 14.3 and 54.1 ± 19, respectively. Respect for patient privacy and respectful communication were desirable, while patients’ autonomy was not desirable. We found a significant direct relationship (*p* = 0.006) between the severity of futile care and respect for patient dignity, with every unit increase in futile care, a 0.01 unit increase was available in patient dignity. We observed no significant association between frequency of futile care and dignity.

**Conclusion:**

Our results indicated the effect of futile care on nurses’ respect for patient dignity. Nurses must raise their awareness through participating in training classes and specialized workshops to improve the level of care, the quality of care, and respect for patient dignity.

## Introduction

The abundance of technological resources at intensive care units increases the lifespan of critically ill patients, but they can lead to futile medical care [1]. According to some studies, 40–60% of the care provided in the ICU is futile [2]. Futile means to be incapable of producing any useful result [3]. In contrast to palliative care, futile medical care is the provision of care to a patient when there is no reasonable hope or chance of benefit. Palliative care helps patients relieve their symptoms or increase their quality of life without increasing lifespan or treating the disease [4]. Patients in the final stages of life experience pain and drug side effects due to futile medical care that imposes great costs on society and interferes with the care of other patients [5]. Nurses providing futile care in special care departments are at risk of job burnout that affects the quality of care and increases staff turnover in these departments [6].

The definition of futile care depends on the patient’s condition and healthcare providers’ personal values, views on life, moral beliefs, and judgments about successful and unsuccessful treatment [7]. Social, occupational, organizational, and individual factors, sense of guilt, grief, fear of legal action and concerns about the patient family's response, all contribute to the futile care [8]. According to Polakala et al. (2017), dealing with moral or legal issues was a challenge for healthcare providers; although, 62 percent believed that scientific knowledge influenced their decisions about futile care [9]. Studies suggest that futile care at the end of life can injury patients and cause moral distress in healthcare workers [10]. Healthcare staff and doctors must observe the principles of medical ethics, including beneficence (i.e. acting in the patient's interest), non-maleficence, autonomy, and justice [11]. Harm refers to physical, moral, and psychological injury [12], while non-abuse is the principle of avoiding unnecessary injuries. Although, some medical interventions may cause pain or harm, the harm can be justified if the benefit of the medical intervention is greater than the harm to the patient and the intervention is not intended to harm the patient. To comply with this principle, healthcare professionals must know their roles and responsibilities in end-of-life care [13]. Improving the care of dying patients is an ongoing clinical priority, but there are concerns about shortcomings in current practice [14]. End-of-life care includes managing pain and symptom, addressing cultural issues, supporting patients and their families at the end of life, experiencing loss, and making ethical decisions [15]. The main priority at the end of life is to have a nice life and a good death. Patients' perceptions of the dignity and meaning of life can help them prepare for death [16]. Pitanupong et al. (2021) found that cancer patients in end-of-life care wanted to receive the full truth about their disease rather than experience any distressing symptoms, were mentally aware in the last hour of life, felt meaningful in life, and could pass away with their loved ones around [17]. According to studies, half of admitted patients experience "loss of dignity" during futile care, with patients in intensive care units being at a higher risk [18].

Dignity is defined as human ability to choose over actions [19]. Some characteristics, such as humanity, are inherent and cannot be taken away; this is a kind of inherent dignity [20]. Respect for dignity is essential as a patient-centered approach with the aim of improving physical, mental, social and spiritual status [21]. Illness, disability, need, loss of power and authority, lack of privacy, treatment and hospitalization all have a negative impact on human dignity [22]. Human dignity is the essence of patient care that is based on humanistic values ​​and respect for the integrity of human beings and their beliefs. With a holistic approach to human beings, this concept covers all stages of illness and final stages of life [23].

Amanda et al. (2018) demonstrated that staff in intensive care units did not respect dignity of patients in the final stages of life [24]. Another study indicated that negative attitudes and non-participation in care decisions compromised the dignity of ICU patients [25]. According to Moen et al. (2015), helplessness and the need for care, inability to speak, and not treating patients as humans all contributed to an inhibition of dignity [26].

Iran is a religious country, with Muslims constituting the majority of the population. Islam provides the spiritual, moral, and social contexts and framework for life, death, and the end of life, and the principles of Islam used in healthcare decisions and end-of-life policies include "no hardship," "necessity," and "no harm" [27]. With the approval of the nursing code of ethics in 2010, ethical principles, such as the preservation of care dignity have become more important in Iran [28].

To reduce futile care and its effects, we must take measures to improve the quality of care and remove obstacles. Managers should manage unnecessary care through setting communication strategies, increasing knowledge and promoting laws, and drafting emotional and moral support systems [5].

According to review of the literature, futile care leads to emotional exhaustion and job burnout [6], and patient suffering [5]. ICU patients are unable to interact, make decisions, choose and participate in treatment, which can have a negative impact on their dignity [26]. This study can help understand futile medical care and the respect for dignity of ICU patients. No study examined the relationship between futile care and patients' dignity from the perspective of nurses. Therefore, this study aimed to investigate the relationship between futile care and patient dignity from the perspectives of nurses working in intensive care units of medical centers in southeastern Iran in 2021.

## Method

This cross-sectional study was conducted in Kerman, southeastern Iran from early May to late July 2021.

### Inclusion and exclusion criteria

The inclusion criteria included the nurses working in intensive care units [29], having at least a bachelor's degree in nursing [30], and full-time employment in the ICU [31]. The exclusion criteria were moving from the intensive care unit (ICU) to other wards of the hospital and failing to answer more than one third of the questions. It is necessary to mention that, inclusion criteria was employment in the ICU department. If they don't answer more than a third of the questions, they will be removed according to the number of questions that will be missed and will affect the results.

### Sample and sampling

This study was conducted on 160 nurses working in adult ICUs in hospitals affiliated with Kerman University of Medical Sciences, and they were included by census method. The study sample was 190 nurses, and finally 160 nurses completed the questionnaire. The study response rate was 0.842.

### Instrument

The researcher collected the study data; she distributed the questionnaires at the beginning of the shift and collected them at the end of the shift so that the nurses had enough time to answer.

The research instrument consisted of three parts: A: Demographic information, B: futile care questionnaire, C: patients’ dignity questionnaire.

- A- Demographic and background information: It included age, sex, level of education, marital status, position, work experience in nursing, work experience in intensive care unit, average working hours per week, participation in training courses, type of shift work.
- B. Nurses’ perceptions of futile care: This 17-item questionnaire was developed by Borhani et al. (2015) and examined intensity and frequency of futile care perceived by nurses. It is based on Corley’s moral distress scale and a review of literature. The questionnaire focuses on good death, pain and discomfort management, treatment discontinuation, effective communication with family members, disagreements among healthcare workers, family members’ disagreements over treatment options, and resource allocation. The questionnaire was scored on a 6-point scale ranging from never (0) to often (5). Using the internal correlation coefficient, ten experts determined its validity to be 82% and its reliability (Cronbach's alpha) to be 85% [30].
- C- Respect for patients’ dignity from the perspective of nurses: It was developed by Raee (2016) using library studies that included 44 questions about respect for patients’ dignity. Nineteen questions were about patient privacy, 10 questions about respectful communication, and 15 questions about autonomy. Items were graded on a Likert scale, including always (3), most of the time (2), sometimes (1) and never (0). Sentences containing the concept of inappropriate dignity were scored in reverse, and nurses who did not have a choice used the option “not applied”. As this option had no score, it was not included in the statistical analysis, but in order to facilitate the analysis of the results, in addition to the mean and score of each domain, this score was also calculated as a percentage. The quality of the factors was investigated by dividing them into four parts and putting indicators in four scales (unfavorable, relatively unfavorable, relatively favorable, and favorable). According to these scales, a score of less than 25% was considered undesirable; a score of 25–50% was considered relatively undesirable; a score of 50–75 percent was considered relatively desirable; and a score of more than 75% was considered desirable. The instrument’s scientific validity has been established through face and content validity, as well as the opinions of 14 faculty members of Isfahan school of nursing and midwifery. All CVR values exceeded 0.51 and CVI values exceeded 0.79. The instrument’s reliability was 0.92 when the Cronbach's alpha coefficient was calculated [32].

### Data analysis

First, the researcher entered data into the SPSS25 and analyzed them after cleaning and coding. Kolmogorov–Smirnov test was used to determine the data normality (Pearson coefficient was calculated for normal distribution, while Spearman's correlation coefficient was calculated for non-normal distribution.).

Descriptive (frequency, percentage, mean and standard deviation) and inferential statistics (Spearman correlation coefficient, Mann–Whitney and Kruskal–Wallis tests) were used. The significance level was 0.05. Unadjusted and multivariate-adjusted linear regression analyses were performed to investigate the effect of futile care on the patient dignity. We used the unadjusted linear model to determine if patient dignity (the response variable) has any relationship with other research variables. All variables with a *p* -value of less than 0.2 were included in the adjusted linear model. Eventually, the final model was developed using the stepwise method.

## Results

The mean age of participants was 30.6 ± 6.9 years. Most of the participants (77.6%) were female and married (62.7%). About 90.6% of them had a bachelor's degree with 7.02 ± 5.7 years of work experience. In addition, the average working hours were 56.8 ± 65.5 h per week. The level of education had a significant relationship with the intensity and frequency of futile care (*p* = 0.01). We found a significant association between shift work and frequency of futile care (*p* = 0.007), as well as between respect for patient dignity, work experience (*p* = 0.02), and working hours per week (*p* = 0.01) (Table 1).

**Table 1:** Relationship between demographic variables, futile care, and respect for patient dignity

Variable	Total = 160	Futile care		Respect for the patient dignity
		Severity	Frequency	
Age	30.6 ± 6.9	Rs = 0.03,*p* = 0.69	Rs = 0.09, *p* = 0.29	Rs = 0.1, *p* = 0.17
Gender				
Male	35 (22.4)	58.5 ± 13.7	55 ± 19.3	3.4 ± 0.6
Female	121 (77.6)	58.3 ± 15.04	54.4 ± 18.4	3.5 ± 0.5
Test result		0.97	0.94	0.16
Marital status				
Single	59(37.7)	59.9 ± 14.9	54.4 ± 20.1	3.5 ± 0.6
Married	99(62.7)	57.1 ± 14.3	54.06 ± 18.3	3.5 ± 0.5
Test result		0.08	0.81	0.71
Ward				
ICU	99(63.5)	59.2 ± 14.8	54.5 ± 20.09	3.4 ± 0.6
Corona ICU	57(36.5)	56.9 ± 14.5	53.9 ± 17.9	3.6 ± 0.5
Test result		0.83	0.68	0.07
Education				
Bachelor’s	144(90.6)	57.3 ± 14.6	53.1 ± 19	3.5 ± 0.5
Master’s	15 (9.4)	65.3 ± 12.3	62.9 ± 17.7	3.6 ± 0.6
Test result		0.01	0.01	0.59
Type of employment				
Committed^1^	66(42.9)	59.1 ± 14.2	51.8 ± 21.6	3.5 ± 0.5
Contract recruiter^2^	63(40.9)	57.7 ± 14.3	56.2 ± 16.1	3.5 ± 0.5
Contract recruiter^3^	25(16.2)	61.9 ± 9.06	59.5 ± 15.5	3.4 ± 0.6
Test result		0.56	0.32	0.79
Work experience	7.02 ± 5.7	Rs = 0.003,*p* = 0.96	Rs = 0.06, *p* = 0.46	Rs = 0.18, *p* = 0.02
Work experience in ICU	4.8 ± 4.9	Rs = 0.08, *p* = 0.38	Rs = 0.02, *p* = 0.82	Rs = 0.13, *p* = 0.15
Training course completion				
Yes	54 (36.7)	60.7 ± 13.2	56.4 ± 19.8	3.5 ± 0.6
No	93 (63.3)	57.3 ± 15.5	54.5 ± 17.2	3.5 ± 0.5
Test result		0.56	0.3	0.33
Working hours per week	56.8 ± 65.5	Rs = 0.11, *p* = 0.21	Rs = 0.007,*p* = 0.94	Rs = -0.23, *p* = 0.01
Position				
Nurse	109 (85.8)	58.6 ± 13.6	54.8 ± 18.5	3.5 ± 0.6
Anesthesia nurse	18 (14.2)	62.8 ± 12.7	54.2 ± 22.9	3.5 ± 0.6
Test result		0.67	0.97	0.45
Shift work				
Fixed	16 (12.4)	51.6 ± 10.9	43.07 ± 18.8	3.3 ± 0.7
In rotation	113 (87.6)	60.5 ± 12.3	57.04 ± 17.3	3.5 ± 0.5
Test result		0.05	0.007	0.11
Number of beds per shift	3.3 ± 3.8	Rs = 0.02, *p* = 0.78	Rs = 0.02, *p* = 0.81	Rs = 0.05, *p* = 0.58

1­ It is obligatory to work for government for two years at a lower rate of pay

2­ Annually contracted with payment similar to hired nurses

3­ Annually contracted with payment less than hired nurses. (In contract employment, a contract is signed with the nurse for at least one year)

We used Mann–Whitney test and Spearman correlation coefficient to examine the relationships and linear regression model to control the effect of confounding variables. Regarding respect for patient dignity, we included variables with *p*-values less than 0.2 into the model after collinear analysis. By controlling the variables of ward, work experience, and working hours per week, severity of futile care had a significant relationship with the respect for patient dignity (*p* = 0.006), so that with every unit increase in futile care score, we found a 0.01 unit increase in the score of patient dignity. However, frequency of futile care had no significant relationship with the respect for dignity (*p* = 0.06) (Table 2).

**Table 2:** Univariate and multivariate linear regression model to investigate the effect of futile care on respect for patient dignity by controlling confounders

	Variable	Β (CI 95%)	SE	*p*-value
The first model	Severity	0.01 (0.004, 0.02)	0.005	0.006
	Ward	0.14 (- 0.08, 0.38)	0.11	0.2
	Work experience	0.02 (0.006, 0.04)	0.02	0.01
	Working hours per week	0 (- 0.001, 0.002)	0.001	0.84
Second model	Frequency	0.006 (0, 0.01)	0.003	0.06
	Ward	0.15 (- 0.1, 0.42)	0.13	0.24
	Work experience	0.02 (- 0.003, 0.04)	0.01	0.08
	Working hours per week	0 (- 0.002, 0.002)	0.001	0.88

The mean scores of patient privacy, patient autonomy, and respectful communication were 2.3 ± 0.4, 2.2 ± 0.6, and 2.2 ± 0.4, respectively. Patient privacy and respectful communication were desirable, while patient autonomy was undesirable. The mean scores of intensity and frequency of futile care were 57.2 ± 14.3 and 54.1 ± 19, respectively (Table 3).

**Table 3:** Examining the scores of respect for the patient dignity and futile care and their dimensions

Variable	Dimensions	Mean ± SD	Undesirable	Relatively undesirable	Relatively desirable	Desirable	Overall status (based on total mean
Respect for patients’ privacy	Physical privacy	2.4 ± 0.5	1.9	5.6	21.9	70.6	desirable
	Confidentiality and privacy	2.5 ± 0.6	2.5	7.5	23.8	66.3	desirable
	Paying attention to trimness and attire	2.4 ± 0.6	0.6	13.8	26.9	58.8	desirable
	Compliance plan	2.08 ± 0.4	0.0	13.8	64.4	21.9	Relatively desirable
	Total	2.3 ± 0.4	0.6	5.0	31.3	63.1	Desirable
Patient autonomy	Giving patients the required information	2.3 ± 0.6	0.6	11.9	25.6	61.9	desirable
	Maintaining autonomy and giving patients the right to choose	2.06 ± 0.6	3.8	29.1	28.5	38.6	Relatively desirable
	Total	2.2 ± 0.6	0.6	15.0	38.1	46.3	Relatively desirable
Respectful communication	Respect for the patient	2.5 ± 0.5	2.5	3.2	23.4	70.9	Desirable
	Nurse-patient relationship	2.3 ± 0.6	2.5	13.4	28.0	56.1	desirable
	Addressing patients politely	1.9 ± 0.6	1.3	51.6	15.5	31.6	Relatively desirable
	Total	2.2 ± 0.4	0.6	7.0	36.7	55.7	Desirable
Respect for patient dignity		2.2 ± 0.4	0.0	5.0	36.3	58.8	Desirable
Futile care	Severity	57.2 ± 14.3	-	-	-	-	
	Frequency	54.1 ± 19	-	-	-	-	

## Discussion

This study aimed to investigate the relationship between futile care and respect for patient dignity from the perspectives of nurses working in intensive care units of medical centers affiliated with Kerman University of Medical Sciences. According to the results, severity of futile care had a significant relationship with respect for the patient's dignity, but frequency of futile care had no significant relationship with respect for dignity. Aghabarary et al. (2016) reported that the value system of doctors and patients, medical goals and socio-cultural and religious context, emotions, and individual characteristics all affected medical futility [33]. Papastavrou et al. (2016) classified factors affecting patient dignity into five groups: (A) patient preferences, verbal abuse, and treating the patient as a unique individual, (B) privacy and confidentiality, C) loss of autonomy and need for help, (D) discrimination, and (E) attribution and reciprocity [34]. According to present results, the mean severity and frequency of futile care were 57.28 ± 14.3 and 54.14 ± 19, respectively, and they found a significant relationship between the level of education, the severity, and frequency of futile care, as well asbetween shift work and frequency of futile care. Mohammadi et al. (2015) showed that the mean scores of nurses' perceptions of futile care severity and frequency were 0.46 ± 3.2 and 1.2 ± 3.7, respectively. They observed a significant relationship between the mean scores of futile care, age, years of work experience, and type of unit [35], with higher levels of education and attendance at training courses leading to a more positive perception of this phenomenon among physicians and nurses. Kadooka et al. (2014) showed that nurses were reluctant to offer potentially futile treatments and emphasized the patient's quality of life; eighty-five point four percent of them experienced futile treatments. Reasons for futile care included patient-related factors, as well as healthcare teams (physicians) [36]. Ruth D et al. (2014) found that nurses provided higher levels of futile care than senior and young physicians did, and that nurses and senior physicians were more anxious about perceived futile care than young physicians were [37]. Asayesh et al. (2018) found that 72.7 percent of the intensive care nurses had a moderate to high level of perception of futile care severity and frequency [37]. Hajilo et al. (2020) showed that most of the participants had a moderate perception of the severity and frequency of futile care. They revealed a significant relationship between moral sensitivity, work experience, and futile care frequency, as well as between age, work experience of nurses in critical care units, and futile care frequency [38]. Rostami et al. (2017) demonstrated that most of the nurses (65.7%) had a moderate perception of futile care; they indicated a significant relationship between average working hours per week and the perception of futile care [39]. Rezaei et al. (2018) reported that the mean score of nurses' perception of futile care was 78.46 ± 14.4 that was more than that of physicians (74.91 ± 12.3) [40]. Moaddaby et al. (2021) showed that the mean score of nurses’ perception of futile care was 63 ± 7; only nurses with a master's degree had higher mean scores in providing futile care, and according to the age group of nurses, futile care was different in socio-cultural contexts [41]. Our results suggested that respect for patients' privacy and respectful communication were desirable, while the patient’s autonomy was undesirable; we observed a significant association between respect for patient dignity, work experience, and working hours per week. Rayat dost et al. (2018) showed no significant difference in respect for the patient dignity among nurses; respect for the patient dignity and its domains, patient privacy, respectful communication, and patient autonomy were desirable or relatively desirable [42]. According to Karimi et al. (2019), more than 60% of the older adults believed that dignity was very important and that respect for dignity was at a good level. Nurses’ work experience had a positive and significant correlation with perception of the importance of dignity for the older adults. Female nurses also cared more about the dignity of the older adults than male ones [43]. Experienced and older nurses paid more attention to patients' autonomy and made better decisions about moral problems compared with nurses with less work experience. Raee et al. (2017) revealed that nurses were more satisfied with patient autonomy and respectful communication that had the highest mean (0.53 ± 2.43, 0.35 ± 2.43) and score, respectively (82%, 79%); patients' privacy had the lowest mean (0.52 ± 2.43, 76%) [44]. Torabizadeh et al. (2021) showed that nurses and patients had different views on respect for dignity. Our results suggested lack of respect for patient dignity, especially in the areas of autonomy and communication. We found no relationship between the mean age of nurses, patients, nurses' work experience, and respect for the patient's dignity, as well as between nurses' gender, marital status, level of education, participation in ethics workshops, and their perception of dignity [45]. Ferri et al. (2015) showed that dignity was not in accordance with the expectations of patients; nurses protected the patient privacy during medical procedures rather than information and verbal communication. Listening to patients' perspectives that they believe are important for their dignity can be helpful in this process [46]. We are unable to increase the lifespan of those in their last days, but when they feel supported and cared for, their last stage of life can be full of meaning and families and caregivers should use all facilities to make the patients' end of lives meaningful. The purpose of palliative care is to provide physical, mental, spiritual and social comfort and relaxation, as well as to solve the problem of meaning that is one of the characteristics of human life. Palliative care in the final moments supports the course of the disease that leads to death, respects patients to have a natural death, and intends neither to.

## Conclusion

Our results showed that the severity of futile care had a significant relationship with respect for patient dignity meaning that futile care played a role in maintaining patients' dignity. Professional ethics education and palliative care can change useless nursing care to effective care and lead to more respect for patients' dignity.

### Strength

To the best of our knowledge, this is the first study that examined the relationship between futile care and patient dignity from the perspectives of nurses working in intensive care units. Nurse Managers and nurses can use our results to become more familiar with futile care and respect for the patient dignity and provide better quality care.

### Limitation

One of the limitations of this study was that we conducted this study on nurses in public hospitals of a specific region in Iran. In order to investigate the relationship between futile care and respect for patient dignity, we require further studies in other areas and cultures, as well as in private hospitals and other wards.

## Data Availability

Data are available by contacting the corresponding author.
